# Association Between Hyponatremia and Rehabilitation Outcomes After Stroke: A Single-Center Retrospective Cohort Study

**DOI:** 10.3390/jcm15135087

**Published:** 2026-06-30

**Authors:** Donghyun Shin, Jongkyu Kim, Songi Han, Sujeong Choi, Eun Sang Yoon

**Affiliations:** Department of Physical Medicine and Rehabilitation, Seoul Medical Center, Seoul 02053, Republic of Korea; lemonshin91@gmail.com (D.S.); onesong@seoulmc.or.kr (S.H.); 2csj@seoulmc.or.kr (S.C.); terryyoon0811@gmail.com (E.S.Y.)

**Keywords:** hyponatremia, stroke rehabilitation, functional status, activities of daily living, length of stay

## Abstract

**Background/Objectives:** This study evaluated the association of hyponatremia with functional outcomes and hospital stay length in patients undergoing acute stroke rehabilitation. **Methods:** We retrospectively reviewed patients undergoing acute stroke rehabilitation. Hyponatremia was identified based on serum sodium levels during hospitalization. After 1:1 propensity score matching (PSM) for age and sex, functional outcomes (Functional Ambulatory Category [FAC], Korean version of the Modified Barthel Index [K-MBI]) and hospital stay were compared between the two groups. Multivariable logistic regression, adjusted for initial NIHSS and baseline FAC/K-MBI scores, was performed to evaluate the independent association of hyponatremia. **Results:** A total of 141 patients with stroke were analyzed. After 1:1 PSM, 50 patients were included in each group. After matching, the hyponatremia group had higher baseline NIHSS and poorer functional status than the non-hyponatremia group. The hyponatremia group also had significantly longer hospital stays (52.70 ± 24.31 vs. 40.92 ± 17.48 days; *p* = 0.007) and lower rates of independent ambulation (16% vs. 44%; *p* = 0.002) and good functional outcomes (22% vs. 56%; *p* < 0.001). After adjusting for baseline NIHSS and functional status, hyponatremia remained independently associated with lower likelihoods of achieving independent ambulation (adjusted OR [aOR], 0.249; 95% CI, 0.064–0.966; *p* = 0.044) and good functional outcomes (aOR, 0.283; 95% CI, 0.086–0.929; *p* = 0.037). **Conclusions:** Hyponatremia was associated with a lower likelihood of achieving independent ambulation and good functional outcomes after rehabilitation, and with a longer hospital stay in patients with acute stroke.

## 1. Introduction

Hyponatremia is a common electrolyte imbalance that occurs following brain injury [1].

Although the mechanisms underlying hyponatremia in traumatic brain injury (TBI) are well described [2], patients with acute stroke are also highly susceptible. Hyponatremia is frequently attributed to stroke-induced neuroendocrine disruptions, such as the syndrome of inappropriate antidiuretic hormone secretion (SIADH) and cerebral salt wasting (CSW) [3,4].

Although several studies have reported that hyponatremia occurs in approximately 11–35% of stroke patients, it has been underinvestigated in the literature [5,6].

Hyponatremia can cause both systemic and neurological complications that may negatively affect functional recovery via several mechanisms. Mechanistically, electrolyte imbalance can induce central nervous system symptoms such as reduced consciousness, fatigue, delirium, and an increased risk of seizures. Furthermore, hyponatremia can directly induce gait instability and aggravate poor nutritional status, collectively delaying participation in rehabilitation and ultimately worsening functional outcomes [7]. Based on these potential mechanisms, we hypothesized that hyponatremia would negatively affect the achievement of good rehabilitation outcomes, prompting a review of the existing literature.

Several studies have reported that patients with acute stroke and hyponatremia tend to have poorer clinical outcomes. However, these studies did not directly evaluate the association between hyponatremia and functional recovery. These studies focused on mortality, neurological severity, or complications following thrombolytic therapy or endovascular intervention rather than functional recovery [8,9]; moreover, few studies have examined the association between hyponatremia and rehabilitation outcomes. A previous study compared functional outcomes before and after a rehabilitation program according to the presence of hyponatremia but only assessed sodium levels at admission [10].

Only a few studies have reported an association between hyponatremia and hospital stay [11]. Thus, this study investigated stroke patients receiving inpatient rehabilitation, utilizing a propensity score-matched design to control for baseline selection bias.

This study aimed to evaluate the independent impact of hyponatremia on functional outcomes at discharge. Additionally, we investigated the association between hyponatremia and the length of hospital stay.

## 2. Materials and Methods

### 2.1. Study Design

This was a single-center retrospective study. The medical records of patients who were diagnosed with acute stroke at Seoul Medical Center, Seoul, Republic of Korea, from March 2021 to February 2024 were obtained. All included patients were diagnosed with acute hemorrhagic or ischemic stroke and received acute management. After acute stroke management, the patients were transferred to the Department of Rehabilitation Medicine for comprehensive acute-phase rehabilitation. Medical records, including sex, age, stroke type, National Institutes of Health Stroke Scale (NIHSS) score, functional status, and various laboratory findings, were collected.

The Institutional Review Board (IRB) of the Seoul Medical Center (2025-10-006) approved this study and waived the requirement for informed consent. This study adhered to all Strengthening the Reporting of Observational Studies in Epidemiology (STROBE) guidelines and reported the required information accordingly (Supplementary Table S3).

### 2.2. Participants

All included patients were diagnosed with acute stroke (hemorrhagic or ischemic), received acute management, and were subsequently transferred to the Department of Rehabilitation Medicine for comprehensive inpatient rehabilitation. Patients aged <18 years with renal dysfunction, pre-diagnosed chronic kidney disease, and those undergoing hemodialysis were excluded.

Patients with a history of stroke, other brain injuries, or those with insufficient rehabilitation (due to unstable medical conditions that inhibit rehabilitation, multidrug-resistant infections, or severe psychological conditions) were also excluded. Insufficient rehabilitation was defined as receiving combined physical and occupational therapy less than one hour a day in the therapy room. Patients with a prolonged hospital stay for non-medical reasons, specifically defined as administrative or sociolegal delays in locating a transfer facility or those with incomplete medical records, were excluded. Incomplete medical records were defined by the absence of key variables required for analysis, such as baseline functional scores (Functional Ambulatory Category [FAC] and Korean version of the Modified Barthel Index [K-MBI]) or serum sodium levels. All the exclusion criteria were applied before the classification of hyponatremia.

### 2.3. Hyponatremia and Data Collection

Hyponatremia was defined as a serum sodium level < 135 mmol/L. When hyponatremia was observed, serum osmolarity was assessed to confirm true hyponatremia and exclude pseudohyponatremia [12]. Routine blood samples were collected at the initial emergency department visit and 1–2 times per week during both acute stroke management in the neurology department and the rehabilitation period.

During hospital stay, patients who showed serum sodium level < 135 mmol/L and low serum osmolality at least once were categorized as the “hyponatremia group”, whereas all others were classified as the “non-hyponatremia group”.

To describe the patients’ baseline characteristics, demographic information (age and sex), stroke-related variables (stroke subtype and NIHSS score), functional status (FAC and K-MBI at transfer), and laboratory findings including estimated glomerular filtration rate (eGFR) and serum albumin levels measured at the time of transfer to the Department of Rehabilitation Medicine were obtained. eGFR was calculated using the Chronic Kidney Disease Epidemiology Collaboration equation. Serum albumin levels were used as indicators of volume-related conditions that may contribute to dilutional hyponatremia.

### 2.4. Functional Status

The NIHSS scores were obtained as the initial severity of neurological injury. Functional status was investigated using the pre-stroke modified Rankin Scale (mRS) to reflect the premorbid functional baseline and the K-MBI and FAC for pre- and post-rehabilitation functional levels. The initial NIHSS score and pre-stroke mRS were evaluated at the time of admission by neurologists, whereas the K-MBI and FAC scores were evaluated by physical and occupational therapists as part of routine clinical care.

Given the retrospective nature of the study, the clinical assessors were not blinded to the serum sodium status during routine evaluations. To prevent bias during data collection, the investigators extracted and documented all functional status data before reviewing serum sodium levels, thereby maintaining investigator blinding during the initial data collection stage. Independent ambulation was defined as FAC ≥ 4 [13]. A good functional outcome was defined as K-MBI ≥ 75 according to a previous rehabilitation study [14,15].

### 2.5. Rehabilitation Protocol

The inpatient rehabilitation protocol was provided 5 days per week (Monday to Friday) as individual sessions in the rehabilitation room. It consisted of physical therapy (60 min, twice daily) and occupational therapy (30 min, twice daily, including dysphagia therapy, when indicated). Speech therapy was administered 1–2 times per week (30 min per session) on an as-needed basis. The patients were transferred to the Department of Rehabilitation Medicine upon achieving medical stability. The standard duration in the rehabilitation department was 4 weeks, unless unavoidable reasons delayed discharge or transfer. All patients underwent rehabilitation based on the same standardized institutional rehabilitation protocol.

### 2.6. Statistical Analysis

The hyponatremia and non-hyponatremia groups were matched using propensity scores. To preserve the statistical power, given the limited sample size, propensity score matching was restricted to age and sex as baseline covariates. Multiple clinical variables, such as stroke severity and baseline functional status, were not strictly matched because this could significantly reduce the number of matched pairs. Instead, these variables were rigorously controlled as covariates in multivariable logistic regression models.

Matching was performed using the nearest-neighbor method in a 1:1 ratio without replacement, with the caliper width set to 0.2 of the standard deviation of the logit of the propensity score [16]. After matching, standardized mean differences (SMDs) for all baseline characteristics were compared to evaluate the adequacy of the matching balance. SMDs were calculated using Cohen’s *d* for continuous variables and were based on the difference in proportions for categorical variables. Multivariable binary logistic regression analyses were performed to evaluate the independent association between hyponatremia and functional outcomes. To adjust for baseline stroke severity and functional status, the initial NIHSS score (measured at admission), FAC score, and K-MBI (measured at transfer to the Department of Rehabilitation Medicine) were included as covariates in the regression model.

Adjusted odds ratios (aORs) and 95% confidence intervals (CIs) were calculated to account for the baseline differences that remained after propensity score matching.

All tests were two-tailed, and statistical significance was set at *p* < 0.05. All statistical analyses were performed using IBM SPSS Statistics for Windows (version 25.0; IBM Corp., Armonk, NY, USA).

## 3. Results

### 3.1. Patient Characteristics

**Figure 1:**
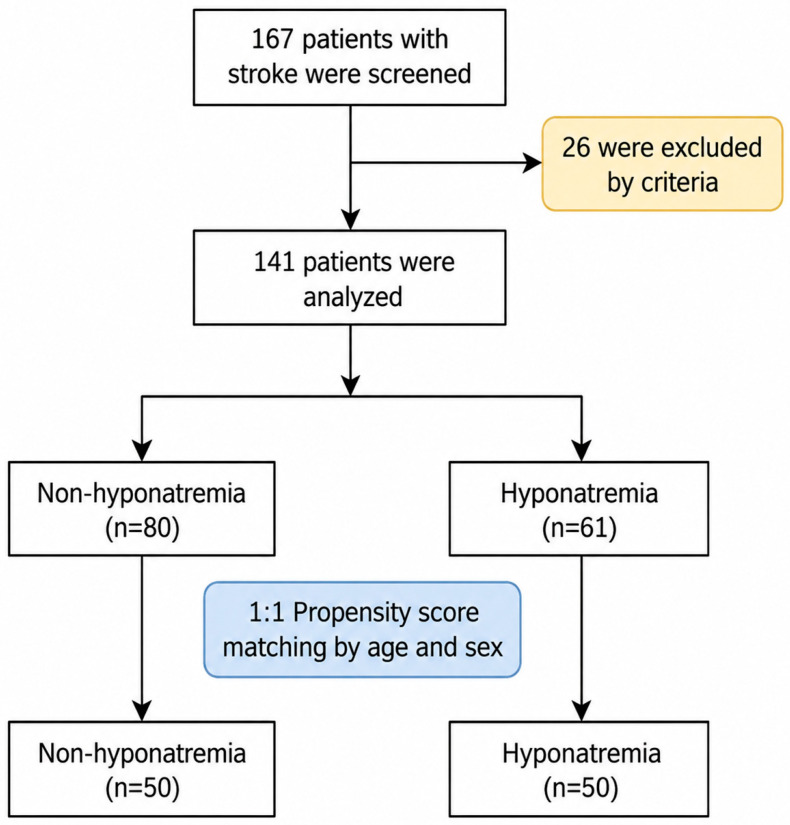
Flowchart of participant screening, exclusion criteria, and follow-up.

A total of 167 patients with stroke were initially screened for this retrospective cohort study. Among them, 26 patients were excluded (detailed reasons for exclusion are provided in Supplementary Table S1), leaving 141 patients as the pre-matched baseline cohort for analysis. The detailed baseline characteristics of this pre-matched cohort are provided in Supplementary Table S2. Sixty-one patients were classified into the hyponatremia group, and 80 were classified into the non-hyponatremia group. After performing 1:1 matching based on age and sex using propensity matching, 50 patients were included in each group for the final analysis (Figure 1).

After matching, the mean age was 67.68 ± 13.67 years in the hyponatremia group and 67.76 ± 13.11 years in the non-hyponatremia group (SMD = 0.006). Each group included 32 male patients (64.00%; SMD = 0.000). Despite matching, substantial baseline imbalances (SMD > 0.5) remained regarding the baseline stroke severity and functional status. Compared with the non-hyponatremia group, the hyponatremia group exhibited higher NIHSS scores at admission (11.50 ± 8.45 vs. 6.76 ± 4.25; SMD = 0.709), lower K-MBI scores at transfer (30.94 ± 23.47 vs. 47.20 ± 29.49; SMD = 0.610), and lower FAC scores at transfer (0.46 ± 0.91 vs. 1.16 ± 1.56; SMD = 0.548) (Table 1).

### 3.2. Functional Outcomes

The length of hospital stay (LOS) was significantly longer in the hyponatremia group (52.70 ± 24.31 days) than in the non-hyponatremia group (40.92 ± 17.48 days). Regarding ambulatory function, the absolute improvement during rehabilitation did not significantly differ between the two groups (ΔFAC, *p* = 0.359); however, the hyponatremia group demonstrated a significantly lower rate of independent ambulation (FAC ≥ 4) at discharge (*p* = 0.002). Similarly, for activities of daily living (ADL), the absolute rehabilitation gain (ΔK-MBI, *p* = 0.225) was statistically comparable between the groups, yet the proportion of patients achieving a good functional outcome (K-MBI ≥ 75) at discharge was significantly lower in the hyponatremia group (*p* < 0.001) (Table 2).

### 3.3. Predictors of Functional Outcomes Adjusted for Baseline Severity and Functional Status

As shown in Table 1, substantial baseline imbalances (SMD > 0.5) were observed after propensity score matching, particularly for the initial neurological severity (initial NIHSS score) and functional status (K-MBI and FAC) at the time of transfer to rehabilitation. To adjust for the confounding effects of baseline clinical severity and initial functional status, multivariable binary logistic regression analyses were performed for each functional outcome. In the regression analysis for independent ambulation (FAC ≥ 4), hyponatremia was significantly and negatively associated with ambulation status at discharge (B = −1.391, *p* = 0.044). The initial NIHSS score was not significantly associated with this outcome (*p* = 0.194), whereas the initial FAC score was a strong positive predictor (*p* < 0.001). Specifically, the adjusted odds ratio (aOR) for achieving independent ambulation in the hyponatremia group was 75.1% lower than that in the non-hyponatremia group (aOR = 0.249, 95% CI: 0.064–0.966). A similar robust pattern was observed for achieving a good functional outcome (K-MBI ≥ 75), where hyponatremia was also significantly and negatively associated with this outcome (B = −1.263, *p* = 0.037). The initial NIHSS score was not statistically significant (*p* = 0.112), whereas the initial K-MBI score demonstrated a highly significant positive association with outcome (*p* < 0.001). The adjusted odds of achieving a good functional outcome in the hyponatremia group were 71.7% lower than those in the non-hyponatremia group (aOR = 0.283, 95% CI: 0.086–0.929).

## 4. Discussion

In the present study, hyponatremia in patients with acute stroke was associated with poorer rehabilitation outcomes and longer hospital stays. Thus, hyponatremia was negatively associated with neurological recovery during neurorehabilitation.

Hyponatremia is common in patients with stroke, with reported incidences of 3.9–45.3% at admission and 40–45% during hospitalization [3]. Water retention or the loss of effective solutes can lead to hyponatremia. Brain injury disrupts water and electrolyte regulation in the body and may cause hyponatremia. This could be attributed to two primary mechanisms. The first is SIADH. Brain injury stimulates the hypothalamic–pituitary axis, causing excessive antidiuretic hormone secretion despite low plasma osmolality. This leads to increased water reabsorption, resulting in dilutional hyponatremia. SIADH clinically presents as euvolemic hyponatremia. The second type is cerebral salt wasting syndrome (CSWS). In this syndrome, brain injury leads to an abnormal increase in natriuretic peptides, such as atrial natriuretic peptide and brain natriuretic peptide, which force the kidneys to excrete sodium. Like sodium, water causes significant volume depletion and clinically presents as hypovolemia [17]. Although SIADH was previously considered the primary cause of hyponatremia following brain injury, recent studies have suggested that CSWS is a more common etiology, specifically in stroke [4]. Other factors such as vomiting, pain, and the use of medications (including diuretics, anticonvulsants, and antidepressants) can also induce hyponatremia [1,3]. Therefore, these mechanisms and their potential causes should be understood in terms of clinical background. Regarding the potential mechanisms through which hyponatremia exerts adverse effects on neurological recovery [7], several physiological pathways may explain this negative association. At the cellular level, significant drops in serum sodium can lead to an osmotic shift of water into the brain cells, exacerbating cerebral edema [18] and secondary brain injury in acute stroke patients [3]. Furthermore, hyponatremia can induce hyponatremia encephalopathy, which manifests as lethargy, confusion, generalized weakness, or cognitive impairment [18].

The association between hyponatremia and the outcomes of patients with TBI has been reported in a systematic review. Ngatuvai et al. [19] have reported that hyponatremia was significantly associated with worse Glasgow Outcome Scale scores at 6 months and a longer length of stay in the intensive care unit.

However, few studies have reported the relationship between hyponatremia and outcomes in patients with stroke. Sah et al. [20] have reported that patients with hyponatremia upon admission had lower initial Glasgow Coma Scale scores and higher mRS scores at discharge. Shima et al. [11] have reported that the hyponatremia group had hospital stays 10.68 days longer than the non-hyponatremia group. However, these studies did not report the rehabilitation outcomes. Our results are consistent with those of previous studies, as hyponatremia was associated with worse functional outcomes and longer hospital stay [11,20].

When specifically evaluating rehabilitation outcomes, Yoshimura et al. [10] reported that Functional Independence Measure improvement was significantly lower in the hyponatremia group during the convalescent phase. Interestingly, our study revealed no significant difference in the net functional gains (ΔFAC, ΔK-MBI) between patients with and without hyponatremia. However, patients with hyponatremia were significantly less likely to achieve K-MBI ≥ 75 and FAC ≥ 4 at discharge. Although the lower likelihood of achieving K-MBI ≥ 75 and FAC ≥ 4 may partly reflect the lower baseline functional status observed in the hyponatremia group (Table 1), our multivariable logistic regression analysis (Table 3) demonstrated that hyponatremia remained independently associated with these outcomes even after adjustment for potential confounders. Thus, both baseline functional impairment and hyponatremia may be associated with poor rehabilitation outcomes. Furthermore, recent rehabilitation frameworks increasingly emphasize multidimensional outcome assessment rather than relying on a single measure of recovery [21]. Recent rehabilitation studies have highlighted the importance of clinically meaningful functionally independent outcomes at discharge [13,14,15]. Therefore, evaluating both continuous functional improvement and clinically meaningful independent outcomes may provide a more comprehensive understanding of rehabilitation success in patients with stroke.

In contrast, Potasso et al. [22] have reported no significant difference in the 3-month functional outcomes between resolved hyponatremia and non-hyponatremia groups. However, their study only measured sodium levels at admission and discharge, which may have missed transient hyponatremia during hospitalization. Additionally, they used the mRS, which is a less sensitive functional evaluation tool than the MBI or FAC, for assessing ADL [14,15]. Serial blood tests were performed at an average period of 46.81 days. We used the K-MBI and FAC instead of the mRS, which could provide a more precise analysis of functional recovery. These factors allowed for a more precise evaluation of the association between sodium levels and functional outcomes and increased the reliability of this study in patients with acute stroke.

This study has some limitations. First, as this was a retrospective single-center study conducted in a public hospital, the results may have been influenced by regional characteristics and specific socioeconomic backgrounds affecting nutritional status. Second, although sodium levels were measured serially, we did not analyze the exact time of onset of hyponatremia. Its association with rehabilitation may differ depending on the timing of its occurrence, which introduces exposure–timing uncertainty and time-dependent bias. In addition, we did not analyze the severity and duration of hyponatremia. Third, we could not determine the specific etiology of hyponatremia or analyze the available data on volume status, urine sodium, urine osmolality, intravenous fluid strategies, or the use of specific medications (e.g., anticonvulsants frequently used in hemorrhagic stroke). Fourth, we did not analyze ischemic and hemorrhagic strokes separately. Fifth, although all patients underwent therapy based on our hospital’s standard rehabilitation protocol (Section 2.5), we did not collect data on the exact dose or actual duration of rehabilitation therapy received by each patient. However, patients who received insufficient rehabilitation were excluded according to the predefined exclusion criteria. Sixth, the application of strict exclusion criteria may have introduced potential selection bias, as 26 patients were excluded from our initial study cohort. Finally, the relatively small sample size limited our ability to achieve an optimal balance between groups after propensity score matching. Consequently, not all baseline variables were perfectly balanced, and the SMD for certain variables increased after matching, indicating the possibility of residual confounding despite our adjustment efforts.

In conclusion, hyponatremia was a common occurrence in the acute phase of stroke and may be negatively associated with good functional outcomes and independent ambulation after rehabilitation. Although our findings support the need for careful monitoring and appropriate management of sodium disturbances in patients with acute stroke, the finding that early correction of hyponatremia directly improved rehabilitation outcomes requires further validation. Well-designed, multicenter prospective studies are necessary to confirm causality and evaluate whether active sodium correction strategies yield better functional recovery.

## 5. Conclusions

Hyponatremia is a common electrolyte imbalance in patients with acute stroke and may be associated with poor rehabilitation outcomes. This single-center retrospective cohort study identified the association of hyponatremia with lower odds of achieving independent ambulation (FAC ≥ 4) and good functional outcomes (K-MBI ≥ 75) at discharge, as well as a longer hospital stay among patients undergoing acute stroke rehabilitation. However, because of residual confounding, baseline functional imbalance, and uncertainty regarding the timing of hyponatremia, prospective studies are needed to clarify whether hyponatremia independently contributes to impaired rehabilitation recovery, and whether its correction improves outcomes.

## Figures and Tables

**Table 1:** Baseline characteristics.

Variable	Non-Hyponatremia (*n* = 50)	Hyponatremia (*n* = 50)	SMD
**Demographics**			
Age (years)	67.76 ± 13.11	67.68 ± 13.67	0.006
Sex, male	32 (64.00)	32 (64.00)	0.000
**Stroke type**			
Cerebral infarction	35 (70.00)	28 (56.00)	0.293
Cerebral hemorrhage	12 (24.00)	17 (34.00)	0.222
Subarachnoid hemorrhage (SAH)	3 (6.00)	5 (10.00)	0.148
**Stroke severity**			
NIHSS at admission	6.76 ± 4.25	11.50 ± 8.45	0.709
**Comorbidities**			
HTN	36 (72.00)	29 (58.00)	0.297
DM	17 (34.00)	18 (36.00)	0.042
Atrial fibrillation (AF)	3 (6.00)	6 (12.00)	0.211
**Premorbid function**			
Pre-stroke mRS	0.60 ± 1.09	0.76 ± 1.24	0.137
**Functional status at transfer to rehabilitation**			
K-MBI at transfer	47.20 ± 29.49	30.94 ± 23.47	0.610
FAC at transfer	1.16 ± 1.56	0.46 ± 0.91	0.548
**Laboratory findings at transfer to rehabilitation**			
Albumin (g/dL)	4.00 ± 0.45	3.78 ± 0.37	0.534
eGFR (mL/min/1.73 m^2^)	88.72 ± 17.44	92.42 ± 21.82	0.187

NIHSS, National Institutes of Health Stroke Scale; HTN, hypertension; DM, diabetes mellitus; AF, atrial fibrillation; mRS, modified Rankin Scale; K-MBI, Korean version of the Modified Barthel Index; FAC, Functional Ambulation Category; eGFR, estimated glomerular filtration rate; SMD, standardized mean difference. Continuous variables are presented as means ± standard deviations (SDs), and categorical variables as numbers and percentages (*n* [%]). This table presents the descriptive statistics of the baseline characteristics after propensity score matching. Group balance was evaluated using SMDs, and no formal hypothesis testing was performed.

**Table 2:** Comparison of functional outcomes between the hyponatremia and non-hyponatremia groups.

**Continuous Variables**					
**Outcome Variable**	**Non-Hyponatremia** **(*n* = 50)**	**Hyponatremia** **(*n* = 50)**	**Test Statistic**	**95% CI of** **Mean Difference**	***p*-Value**
**ΔFAC (score)**	1.64 ± 1.35	1.32 ± 1.27	1.221 (t)	0.32 [−0.20, 0.84]	0.359
**ΔK-MBI (score)**	19.68 ± 17.93	16.14 ± 20.41	0.921 (t)	3.54 [−4.09, 11.17]	0.225
**LOS (days)**	40.92 ± 17.48	52.70 ± 24.31	−2.781 (t)	−11.78 [−20.20, −3.37]	0.007 *
**Categorical Variables**					
**Outcome Variable**	**Non-Hyponatremia** **(*n* = 50)**	**Hyponatremia** **(*n* = 50)**	**Test Statistic**	**OR [95% CI]**	***p*-Value**
**Independent ambulation** **(FAC ≥ 4) [*n* (%)]**	22 (44)	8 (16)	9.33 (χ^2^)	0.24 (0.10–0.62)	0.002 *^†^ (0.004 *^‡^)
**Good functional outcome** **(K-MBI ≥ 75) [*n* (%)]**	28 (56)	11 (22)	12.15 (χ^2^)	0.22 (0.09–0.53)	<0.001 *^†^ (<0.001 *^‡^)
**ΔK-MBI ≥ 5** **[*n* (%)]**	40 (80)	33 (66)	2.49 (χ^2^)	0.49 (0.10–1.20)	0.115 ^†^ (0.176 ^‡^)
**ΔK-MBI ≥ 10** **[*n* (%)]**	33 (66)	27 (54)	1.50 (χ^2^)	0.60 (0.27–1.36)	0.221 ^†^ (0.310 ^‡^)

FAC, Functional Ambulation Category; K-MBI, Korean version of Modified Barthel Index; LOS, length of hospital stay; CI, confidence interval; OR, odds ratio. Continuous variables are presented as means ± standard deviations, and categorical variables as numbers and percentages (n [%]). ΔFAC and ΔK-MBI represent the changes in FAC and K-MBI scores between admission and discharge. Independent ambulation was defined as FAC ≥ 4, and good functional outcome as K-MBI ≥ 75. *p*-values were obtained using independent t-tests for continuous variables and both Pearson’s chi-square and Fisher’s exact tests for categorical variables. ^†^ Pearson’s chi-square test; ^‡^ Fisher’s exact test. When both tests were applied, p-values are reported as Pearson ^†^ (Fisher ^‡^). Statistically significant at *p* < 0.05, indicated by an asterisk (*).

**Table 3:** Multivariable logistic regression analysis for functional outcomes adjusted for baseline severity and functional status.

Dependent Variable (Outcome)	Predictor Variable	B (β)	SE	Wald	aOR	95% CI for aOR	*p*-Value
Independent ambulation (FAC ≥ 4)	Hyponatremia	−1.391	0.692	4.041	0.249	[0.064, 0.966]	0.044 *
	Initial NIHSS	0.063	0.049	1.684	1.065	[0.968, 1.172]	0.194
	Initial FAC	1.494	0.314	22.640	4.457	[2.408, 8.248]	<0.001 *
Good functional outcome (K-MBI ≥ 75)	Hyponatremia	−1.263	0.607	4.335	0.283	[0.086, 0.929]	0.037 *
	Initial NIHSS	0.086	0.054	2.526	1.090	[0.980, 1.212]	0.112
	Initial K-MBI	0.081	0.017	22.219	1.084	[1.048, 1.121]	<0.001 *

Multivariable binary logistic regression analysis was performed for each dependent variable to evaluate the independent impact of hyponatremia. In the model for independent ambulation (FAC ≥ 4), adjustments were made for baseline stroke severity (initial NIHSS score) and initial walking ability (initial FAC). In the model for good functional outcome (K-MBI ≥ 75), adjustments were made for the initial NIHSS score and baseline functional status (initial K-MBI). Values are expressed as B, SE, Wald statistic, and aOR with 95% CI. Statistical significance was set at *p* < 0.05, as indicated by an asterisk (*).

## Data Availability

Data presented in this study are available upon request from the corresponding author. The data are not publicly available due to privacy and ethical restrictions.

## Supplementary Materials

The following supporting information can be downloaded at: https://www.mdpi.com/article/10.3390/jcm15135087/s1, Table S1: Detailed reasons for patient exclusion at baseline; Table S2: Baseline characteristics of the pre-matched cohort; Table S3: STROBE Statement—checklist of items that should be included in reports of cohort studies.

## Abbreviations

The following abbreviations are used in this manuscript:

ADL	Activities of Daily Living
AF	Atrial Fibrillation
aOR	Adjusted Odds Ratio
B	Unstandardized Coefficient
CI	Confidence Interval
CSWS	Cerebral Salt Wasting Syndrome
DM	Diabetes Mellitus
eGFR	Estimated Glomerular Filtration Rate
FAC	Functional Ambulation Category
HTN	Hypertension
IRB	Institutional Review Board
K-MBI	Korean version of the Modified Barthel Index
LOS	Length of Hospital Stay
mRS	Modified Rankin Scale
NIHSS	National Institutes of Health Stroke Scale
OR	Odds Ratio
SAH	Subarachnoid Hemorrhage
SD	Standard Deviation
SE	Standard Error
SIADH	Syndrome of Inappropriate Antidiuretic Hormone Secretion
SMD	Standardized Mean Difference
STROBE	Strengthening the Reporting of Observational Studies in Epidemiology
TBI	Traumatic Brain Injury
Wald	Wald Statistic
