# Supplementary material for: Association Between Hyponatremia and Rehabilitation Outcomes After Stroke: A Single-Center Retrospective Cohort Study
Source: J Clin Med. 2026 Jun 30;15(13):5087. doi: 10.3390/jcm15135087 (PMC13363066; doi:10.3390/jcm15135087)
Supplement: Supplementary file 1 [file jcm-15-05087-s001.zip › jcm-4302037-supplementary.pdf]

Table S1. Detailed reasons for patient exclusion at baseline.

**Reason for exclusion**	**Number of patients (n = 26)**
Age < 18 years	1
Renal dysfunction	4
Prior neurological disorder	10
Insufficient rehabilitation	8
*- Unstable medical conditions	5
*- Multidrug-resistant infections	2
*- Severe psychological conditions	1
Non-medical reason for prolonged hospitalization	1
Incomplete medical records	2
*- Missing initial NIHSS score	1
*- Missing discharge K-MBI score	1

NIHSS, National Institutes of Health Stroke Scale; K-MBI, Korean version of the Modified Barthel Index.

Insufficient rehabilitation was defined as receiving ≤1 h of combined physical and occupational therapy per day in the rehabilitation room due to unstable medical conditions, multidrug-resistant infections, or severe psychological conditions. ***Non-medical reasons for prolonged hospitalization*** include administrative or socio-legal delays in locating a transfer facility. ***Incomplete medical records*** were defined as the absence of key variables required for analysis, such as baseline functional scores or serum sodium levels.

Table S2. Baseline characteristics of pre-matched cohort.

Variable	Non-hyponatremia (n=80)	Hyponatremia (n=61)	SMD
**Demographics**			
Age, year	66.63 ± 13.82	69.61 ± 13.98	0.214
Sex, male	48 (60.00)	37 (60.66)	0.013
**Stroke type**			
Cerebral infarction	54 (67.50)	35 (57.38)	0.210
Cerebral hemorrhage	19 (23.75)	21 (34.43)	0.235
Subarachnoid hemorrhage	7 (8.75)	5 (8.20)	0.022
**Stroke severity**			
NIHSS at admission	8.18 ± 6.52	12.11 ± 9.19	0.494
**Comorbidities**			
HTN	51 (63.75)	38 (62.30)	0.031
DM	21 (26.25)	22 (36.07)	0.213
Atrial fibrillation	7 (8.75)	10 (16.39)	0.231
**Premorbid function**			
Pre-stroke mRS	0.53 ± 0.99	0.75 ± 1.23	0.197
**Functional status at transfer to rehabilitation**			
K-MBI at transfer	47.30 ± 27.83	29.83 ± 23.38	0.680
FAC at transfer	1.05 ± 1.46	0.48 ± 0.98	0.458
**Laboratory findings at transfer to rehabilitation**			
Albumin (g/dL)	4.01 ± 0.45	3.75 ± 0.41	0.604
eGFR (mL/min/1.73 m^2)	89.38 ± 18.62	91.05 ± 22.52	0.081

SMD, standardized mean difference; HTN, hypertension; DM, diabetes mellitus; mRS, modified Rankin Scale; K-MBI, Korean version of the Modified Barthel Index; FAC, Functional Ambulatory Category; eGFR, estimated glomerular filtration rate; NIHSS, National Institutes of Health Stroke Scale.

STROBE Statement—checklist of items that should be included in reports of observational studies

STROBE Statement—Checklist of Items for observational Studies
The STROBE checklist below provides a summary of the reporting items recommended for observational studies. All relevant elements of this manuscript have been addressed in accordance with the STROBE guidelines.

Table S3. STROBE Statement Checklist

	Item No.	Recommendation	Page No.	Relevant text from manuscript
**Title and abstract**	1	(*a*) Indicate the study's design with a commonly used term in the title or the abstract	1	The study design was clearly identified in the title as a "single-center retrospective cohort study": "Association Between Hyponatremia and Rehabilitation Outcomes After Stroke: A Single-Center Retrospective Cohort Study." In addition, the Methods section of the Abstract states that patient records were retrospectively reviewed.
		(*b*) Provide in the abstract an informative and balanced summary of what was done and what was found	1	An informative summary of the background, methods, results (including FAC, K-MBI, hospital stay, adjusted Odds ratios), and conclusions is provided in the Abstract.
**Introduction**				

Background/rationale	2	Explain the scientific background and rationale for the investigation being reported	1-2	The Introduction describes the prevalence and mechanisms of hyponatremia after stroke, its potential association with poor rehabilitation outcomes, and the limited evidence regarding functional recovery and length of hospital stay in patients with acute stroke.
Objectives	3	State specific objectives, including any prespecified hypotheses	2	The study aimed to evaluate the association between hyponatremia and functional outcomes at discharge and length of hospital stay in patients undergoing acute stroke rehabilitation. We hypothesized that hyponatremia would be associated with poorer rehabilitation outcomes.
**Methods**				
Study design	4	Present key elements of study design early in the paper	2	The study was designed as a single-center retrospective cohort study, as described in Section 2.1
Setting	5	Describe the setting, locations, and relevant dates, including periods of recruitment, exposure, follow-up, and data collection	2	The study setting (Seoul Medical Center, Seoul, Republic of Korea), study period (March 2021 to February 2024), and data

				collection procedures are described in Section 2.1.
Participants	6	(*a*) *Cohort study*—Give the eligibility criteria, and the sources and methods of selection of participants. Describe methods of follow-up<br>*Case-control study*—Give the eligibility criteria, and the sources and methods of case ascertainment and control selection. Give the rationale for the choice of cases and controls<br>*Cross-sectional study*—Give the eligibility criteria, and the sources and methods of selection of participants	2-3	Eligibility criteria, exclusion criteria, and participant selection methods are described in Section 2.2.
		(*b*) *Cohort study*—For matched studies, give matching criteria and number of exposed and unexposed<br>*Case-control study*—For matched studies, give matching criteria and the number of controls per case	4-5	Propensity score matching was performed at a 1:1 ratio using age and sex as matching variables. After matching, 50 patients were included in each group, as described in Section 2.6 and Figure 1.
Variables	7	Clearly define all outcomes, exposures, predictors, potential confounders, and effect modifiers. Give diagnostic criteria, if applicable	3	Definitions of hyponatremia, functional outcomes (FAC and K-MBI), baseline characteristics, and clinical variables are provided in Sections 2.3 and 2.4.
Data sources/ measurement	8*	For each variable of interest, give sources of data and details of methods of assessment (measurement). Describe comparability of assessment methods if there is more than one group	3	Clinical variables were obtained from medical records. Definitions and assessment methods for serum sodium levels, NIHSS, FAC, K-MBI, and laboratory variables are described in Sections 2.3 and 2.4.

Bias	9	Describe any efforts to address potential sources of bias	4	To reduce selection bias, propensity score matching was performed. In addition, multivariable logistic regression analyses were used to adjust for baseline differences, including initial stroke severity and baseline functional status, as described in Section 2.6.
Study size	10	Explain how the study size was arrived at	4-5	The study included all eligible patients who met the inclusion and exclusion criteria during the study period. The participant selection process and final study population are described in Section 3.1 and Figure 1.
Continued on next page Quantitative variables	11	Explain how quantitative variables were handled in the analyses. If applicable, describe which groupings were chosen and why	3	Hyponatremia was defined as a serum sodium level <135 mmol/L. Independent ambulation was defined as FAC ≥4, and a good functional outcome was defined as K-MBI ≥75. These definitions are described in Sections 2.3 and 2.4.
Statistical methods	12	(*a*) Describe all statistical methods, including those used to control for confounding	4	Statistical methods, including propensity score matching, comparison of baseline characteristics, and

				multivariable logistic regression analyses, are described in Section 2.6.
		(*b*) Describe any methods used to examine subgroups and interactions	-	N/A (Subgroup and interaction analyses were not performed.)
		(*c*) Explain how missing data were addressed	3	Patients with incomplete medical records were excluded from the study as described in Section 2.2.
		(*d*) *Cohort study*—If applicable, explain how loss to follow-up was addressed<br>*Case-control study*—If applicable, explain how matching of cases and controls was addressed<br>*Cross-sectional study*—If applicable, describe analytical methods taking account of sampling strategy	4	Propensity score matching was performed using a 1:1 nearest-neighbor approach based on age and sex, as described in Section 2.6.
		(*e*) Describe any sensitivity analyses	-	N/A (Sensitivity analyses were not performed.)
**Results**				
Participants	13*	(a) Report numbers of individuals at each stage of study—e.g., numbers potentially eligible, examined for eligibility, confirmed eligible, included in the study, completing follow-up, and analysed	4-5	A total of 167 patients were screened. After applying the eligibility criteria, 141 patients were included in the pre-matched cohort. Following 1:1 propensity score matching, 100 patients (50 per group) were included in the final analysis, as described in Section 3.1 and Figure 1.
		(b) Give reasons for non-participation at each stage	2-3	Reasons for exclusion are provided in Supplementary Table S1, based on the

				eligibility criteria described in Section 2.2.
		(c) Consider use of a flow diagram	5	A flow diagram (Figure 1) is provided to illustrate participant screening, exclusion, matching, and final inclusion.
Descriptive data	14*	(a) Give characteristics of study participants (e.g., demographic, clinical, social) and information on exposures and potential confounders	6	Baseline demographic characteristics, stroke type, comorbidities, functional status, and laboratory findings are summarized in Table 1.
		(b) Indicate number of participants with missing data for each variable of interest	2,4	There were no missing data for the analyzed variables, as patients with incomplete medical records were excluded before analysis (Section 2.2). The participant selection process is described in Section 3.1).
		(c) *Cohort study*—Summarise follow-up time (e.g., average and total amount)	6-7	The observation period corresponded to the inpatient rehabilitation stay. Length of hospital stay (LOS) is reported in Section 3.2 and Table 2.
Outcome data	15*	*Cohort study*—Report numbers of outcome events or summary measures over time	6-7	Numbers and percentages of patients achieving independent ambulation (FAC ≥ 4) and good functional outcomes (K-MBI ≥ 75), as well as changes in

				FAC and K-MBI scores and LOS, are reported in Section 3.2 and Table 2.
		Case-control study—Report numbers in each exposure category, or summary measures of exposure	-	-
		Cross-sectional study—Report numbers of outcome events or summary measures	-	-
Main results	16	(*a*) Give unadjusted estimates and, if applicable, confounder-adjusted estimates and their precision (eg, 95% confidence interval). Make clear which confounders were adjusted for and why they were included	6-8	Unadjusted outcome comparisons are presented in Table 2. Adjusted odds ratios (aORs) with 95% confidence intervals from multivariable logistic regression analyses are presented in Section 3.3 and Table 3. The models were adjusted for baseline stroke severity (initial NIHSS) and baseline functional status (initial FAC or K-MBI).
		(*b*) Report category boundaries when continuous variables were categorized	6-7	Independent ambulation was defined as FAC ≥ 4, and good functional outcome was defined as K-MBI ≥ 75, as described in Section 3.2 and Table 2.
		(*c*) If relevant, consider translating estimates of relative risk into absolute risk for a meaningful time period	7	Absolute numbers and percentages for each outcome category are presented in Table 2.
Continued on next page Other analyses	17	Report other analyses done—eg analyses of subgroups and interactions, and sensitivity analyses	-	N/A (Subgroup, interaction, and sensitivity analyses were not performed.)

**Discussion**				
Key results	18	Summarise key results with reference to study objectives	10	Hyponatremia was associated with lower odds of achieving independent ambulation (FAC ≥ 4) and good functional outcomes (K-MBI ≥ 75) at discharge, as well as longer hospital stay among patients undergoing acute stroke rehabilitation.
Limitations	19	Discuss limitations of the study, taking into account sources of potential bias or imprecision. Discuss both direction and magnitude of any potential bias	10	The limitations of this study are discussed in Section 4 and include the retrospective single-center design, exposure-timing uncertainty, lack of analysis of hyponatremia severity and duration, inability to determine the etiology of hyponatremia, lack of rehabilitation dose data, potential selection bias due to exclusions, and residual confounding despite propensity score matching.
Interpretation	20	Give a cautious overall interpretation of results considering objectives, limitations, multiplicity of analyses, results from similar studies, and other relevant evidence	8-10	The results are interpreted in the context of previous studies and the study limitations. Potential mechanisms of hyponatremia are discussed as clinical background, and the findings are compared with previous

				studies evaluating stroke and rehabilitation outcomes. A cautious interpretation emphasizing association rather than causality is provided in discussion and conclusion.
Generalisability	21	Discuss the generalisability (external validity) of the study results	10	The generalisability of the findings is discussed in Section 4. Because this was a single-center study conducted in a public hospital, the results may have been influenced by regional and socioeconomic characteristics, which may limit external validity.
**Other information**				
Funding	22	Give the source of funding and the role of the funders for the present study and, if applicable, for the original study on which the present article is based	11	The authors declare that this research received no external funding.

*Give information separately for cases and controls in case-control studies and, if applicable, for exposed and unexposed groups in cohort and cross-sectional studies.

Note: An Explanation and Elaboration article discusses each checklist item and gives methodological background and published examples of transparent reporting. The STROBE checklist is best used in conjunction with this article (freely available on the Web sites of PLoS Medicine at http://www.plosmedicine.org/, Annals of Internal Medicine at http://www.annals.org/, and Epidemiology at http://www.epidem.com/). Information on the STROBE Initiative is available at www.strobe-statement.org.
